# Cyanobacteria and Their Metabolites in Mono- and Polidominant Shallow Eutrophic Temperate Lakes

**DOI:** 10.3390/ijerph192215341

**Published:** 2022-11-20

**Authors:** Ksenija Savadova-Ratkus, Hanna Mazur-Marzec, Jūratė Karosienė, Kaarina Sivonen, Suvi Suurnäkki, Jūratė Kasperovičienė, Ričardas Paškauskas, Judita Koreivienė

**Affiliations:** 1 Laboratory of Algology and Microbial Ecology, Nature Research Centre, Akademijos Str. 2, LT-08412 Vilnius, Lithuania; 2 Division of Marine Biotechnology, Institute of Oceanography, University of Gdańsk, M. J. Piłsudskiego 46, PL-81378 Gdynia, Poland; 3 Department of Microbiology, University of Helsinki, Viikinkaari 9, Biocenter 1, P.O. Box 56, FIN-00014 Helsinki, Finland; 4 Nanoscience Center, Department of Biological and Environmental Science, University of Jyväskylä, P.O. Box 35, FIN-40014 Jyväskylä, Finland

**Keywords:** microcystins, saxitoxin, anatoxin-a, non-ribosomal peptides, oligopeptides, *Aphanizomenon gracile*, *Microcystis*, *Planktothrix agardhii*

## Abstract

Monodominant (one species dominates) or polidominant (multiple species dominate) cyanobacterial blooms are pronounced in productive freshwater ecosystems and pose a potential threat to the biota due to the synthesis of toxins. Seasonal changes in cyanobacteria species and cyanometabolites composition were studied in two shallow temperate eutrophic lakes. Data on cyanobacteria biomass and diversity of dominant species in the lakes were combined with chemical and molecular analyses of fifteen potentially toxin-producing cyanobacteria species (248 isolates from the lakes). Anatoxin-a, saxitoxin, microcystins and other non-ribosomal peptides formed the diverse profiles in monodominant (*Planktothrix agardhii*) and polidominant (*Aphanizomenon gracile*, *Limnothrix* spp. and *Planktolyngbya limnetica*) lakes. However, the harmfulness of the blooms depended on the ability of the dominant species to synthesize cyanometabolites. It was confirmed that *P*. *agardhii* produced a greater amount and diverse range of MCs and other NRPs. In the polidominant lake, isolates of the co-dominant *A*. *gracile*, *L*. *planctonica* and *P*. *limnetica* synthesized no or only small amounts of cyanometabolites. In general, the profile of cyanometabolites was greater in cyanobacteria isolates than in environmental samples, indicating a high potential for toxic cyanobacteria bloom.

## 1. Introduction

Cyanobacteria cause harmful blooms worldwide and their occurrence in aquatic ecosystems is increasing in frequency and intensity due to climate change and anthropogenic eutrophication [1,2]. Cyanobacteria usually form monodominant (one dominant species) or polidominant (several dominant species) blooms, especially in sensitive aquatic ecosystems such as shallow eutrophic lakes [3,4]. Some species may form a single dominant perennial bloom, such as *Planktothrix agardhii* [5], while other species may dominate in the community for a particular season or share dominance with other prevailing cyanobacterial species. Thus, cyanobacteria species differ in their ability to persist in a bloom.

Blooms are usually caused by species of the genus *Microcystis*, *Dolichospermum*, *Aphanizomenon sensu lato* and *Planktothrix* in countries along the European continent [6]. These cyanobacteria not only induce blooms, but also synthesize a variety of different cyanometabolites. Typically, up to 40–70% of blooms are reported as toxic [7,8]. Since toxins are the major concern associated with cyanobacteria blooms, it is important to evaluate how the pool of cyanometabolites differs between mono- and polidominant lakes. Bloom toxicity and cyanometabolites profile depend on a variety of factors: the composition of cyanobacteria species and isolates, biomass, the percentage of toxic individuals in the population [9], the ability to produce toxic cyanometabolites [6], the expression of genes responsible for metabolite synthesis [10,11], and abiotic factors such as light, temperature and nutrients. Therefore, seasonal and spatial changes in the composition of the dominant cyanobacteria species and their biomass result in variation in the structure and quantity of cyanotoxins in ecosystems [12]. Thirty years of studies assessing the occurrence and production of cyanotoxins have not fully elucidated the causality of why, when and which species produce toxins alone or in combination with other secondary metabolites [13].

The studies usually address the following topics: cyanometabolites in lakes (cyanotoxins and/or non-ribosomal peptides (NRPs)) and cyanobacteria species dynamic (biomass and/or diversity of bloom-forming dominant cyanobacteria) or cyanometabolites in cyanobacteria isolates. Phytoplankton structure was not provided in a comprehensive survey of cyanotoxins in 137 European lakes [14]; this information would be a valuable contribution to complete profile of potential producers in the continent. Therefore, the joint efforts of a team of experts to carry out a complex study on cyanobacteria and their metabolites, based on the analysis of field samples, isolates of cyanobacteria from the same lakes, and chemical and molecular analyses, are highly needed. Moreover, only the most common cyanotoxin microcystins (MCs) is well documented in European freshwaters [15,16], although other known cyanotoxins such as saxitoxin (STX), cylindrospermopsin (CYN) or anatoxin-a (ATX-a) have been detected and a great diversity of their potential producers may also occur in freshwaters of the continent. Moreover, the known cyanotoxins most likely represent only a small fraction of the bioactive compounds produced by cyanobacteria. Therefore, the assessment of other NRPs, which are also frequently detected in water bodies of different European countries, is equally important [5,17,18,19,20,21]. Among the NRPs (except MCs), the most known compounds in cyanobacteria are cyanopeptolins (CPs)-36%, microginins (MRs)-14%, aeruginosins (AERs)-13%, followed by cryptophycins and anabaenopeptins (APs)-9% [22,23]. For a complete overview of the main cyanobacteria producers and the spectrum of cyanometabolites in lakes, isolates and cyanobacteria population studies are also crucial.

The aim of the study was to assess differences in the composition of cyanobacteria and cyanometabolites in mono- and polidominant shallow eutrophic temperate lakes based on field data and cyanobacteria isolates from the studied lakes, focusing on the species responsible for the synthesis of the toxic compounds that determine the profile of cyanometabolites in aquatic ecosystems.

## 2. Materials and Methods

### 2.1. Study Area

The study was conducted in two shallow eutrophic temperate lakes: Širvys and Jieznas located in the eastern part of Lithuania. The catchment area of Lake Jieznas is dominated by agricultural land, while Lake Širvys is surrounded almost equally by natural biotopes and agriculture (Table 1). Lake Širvys is classified as problematic lake with frequent blooms, while Lake Jieznas is designated as a lake in critical condition with perennial blooms [24]. The lakes are used for recreational purposes and fishing.

### 2.2. Sampling, Physicochemical Data and Phytoplankton Analysis

Sampling was conducted from May to October in Lake Širvys (2014–2015) and Lake Jieznas (2015). Water samples were collected from the surface layer in the deepest part of the lakes using a Ruttner sampler. Water temperature, pH, conductivity, and dissolved oxygen were measured in situ using a F/Set-3 portable multiline meter with selective electrodes. Secchi depth was measured using a Secchi disc. Chlorophyll-*a* (chl-*a*) was measured using a fluorometer AlgaeLabAnalyser (bbe Moldaenke GmbH, Schwentinental, Germany). Total nitrogen (TN) and total phosphorus (TP) were determined according to standard methods (LST ENISO 10304; LST EN ISO 14911).

Surface water samples (1 l volume) were analysed to assess cyanobacteria bloom dynamics and seasonal succession. Samples for analysis were preserved with 4% (*v/v*) formaldehyde solution. Species identification and counting were performed in a Nageotte chamber using a light microscope. At least 600 counting units per sample were estimated [25]. Biomass was calculated using geometric shape formulas described in Olrik et al. [26] and Olenina et al. [25]. Identification of cyanobacteria species was based on morphology according to Komárek and Anagnostidis [27,28], Komárek [29].

### 2.3. Isolation and Maintenance of Cyanobacterial Isolates

Isolates of the predominant potentially toxic cyanobacterial species (15 species, 248 isolates) were isolated from the studied lakes Širvys and Jieznas to determine various cyanometabolites. Monocultures were isolated by the micropipette washing method from the surface water samples collected with a 20 µm mesh plankton net. Cultures were maintained in modified MWC medium [30] at 20 °C, 30 µmol m^−2^ s^−1^ cool white fluorescent illumination, and day-night ratio of 12:12. Isolates were identified based on morphology according to Komárek and Anagnostidis [27,28], Komárek [29]. The isolates were deposited in the culture collection of algae and cyanobacteria of the Nature Research Centre (Lithuania).

### 2.4. Chemical Analysis of Cyanometabolites

Cyanometabolites were analysed in cyanobacterial material from the surface water layer of the lakes and in isolates. Lakes’ water (150–350 mL) was filtered through GF/F filters, cyanobacterial cultures were concentrated by centrifugation at 8000 rpm for 6–12 min and freeze-dried. MCs and other NRPs, ATX-a and CYN were extracted using 75% methanol in MiliQ water. STX was extracted with a mixture of 4 mM ammonium formate buffer (pH 3.5) and acetonitrile (95:5, *v/v*) at a ratio of 2:3. All samples were vortexed for 5 min and held in a bath sonicator (Sonorex, Bandelin, Berlin, Germany) for 5 min. The filters of the field samples were homogenized for 1 min with an ultrasonic homogeniser HD 2070 Sonopuls (Bandelin, Berlin, Germany) before bath sonication. The extracts were centrifuged at 10,000 rpm for 20 min, and the supernatant was transferred to chromatography vials. Analysis was performed using LC-MS/MS (QTRAP5500, Applied Biosystems, Siex; Concorde, ON, Canada) equipped with a turbo ion spray ionization, operating in positive mode using the information–dependent acquisition method (IDA) for the detection of MCs and other NRPs [5]. Quantitative analysis of cyanotoxins was performed by multiple reaction monitoring (MRM) using standards. Quantification of MCs was performed according to Khomutovska et al. [31] and STX was described in Karosienė et al. [32]. Data were analysed using Analyst QS^®^ 1.5.1 software.

### 2.5. Evaluation of the Copy Number of the Microcystin (mcyE) Gene in Field Samples (qPCR)

Phytoplankton biomass collected from the water surface of Lake Širvys was concentrated on GF/F filters and stored at −70 °C. DNA was extracted using the PowerWater^®^DNA Isolation Kit according to the manufacturer’s protocol and stored at −20 °C. Quantification of copy number of the *mcy*E gene was performed by quantitative real–time PCR (qPCR). The PCR reaction mix had a total volume of 25 µL, and contained 5 µL of DNA, 1.25 µL of each primer (300 nM), and 12.5 µL of the ready–to–use reaction mix prepared according to the manufacturer’s instructions. The forward primer mcyE-F2 described in Vaitomaa et al. [33] and the reverse primer mcyE-plaR3 by Rantala et al. [34] were used. PCR was performed in a 96-well skirted PCR plate (4titude) using a device Bio-Rad CFX96. PCR protocol was performed according to Rantala et al. [35] with minor modifications: preincubation at 95 °C, 3 min, denaturation 40 cycles 95 °C, 10 s and annealing at 59 °C, 45 s, melting curve analysis 95 °C, 15 s, 58 °C, 30 s, 95 °C, 5 s. Each environmental sample was run in triplicate, also negative and positive controls, external standard dilutions were performed.

### 2.6. Molecular Analysis of Cyanobacterial Isolates

Genes analysis: *mcy*E and *ana*C. Isolates of *Planktothrix agardhii* and *Dolichospermum* spp. at exponential phase were centrifuged at 8000 rpm for 6–12 min and freezed at −70 °C. DNA was extracted using the E.Z.N.A SP Plant DNA Kit for isolates according to the manufacturer’s instructions. To verify that the samples contained sufficient cyanobacterial DNA, primers CYA359F, CYA781R(a), and CYA781R(b) were used for analysis according to the PCR protocol described in Nübel et al. [36] with minor modifications: 94 °C, 3 min; 35 × (94 °C, 30 s; 56 °C, 30 s; 72 °C, 1 min); 72 °C, 10 min. The specific primers (described in Vaitomaa et al. [33] and Rantala et al. [34]) were used for the detection of *mcy*E in *P*. *agardhii* isolates and *ana*C gene in *Dolichospermum* spp. isolates (according to Rantala-Ylinen et al. [37]). The amplified products were separated on a 1% TAE agarose gel. Conventional PCR was used to determine whether the isolate had the gene responsible for cyanotoxin production (band present) or whether the product was absent (no band). Negative and positive controls were also performed each time.

### 2.7. Statistical Analysis

The relationship between *P*. *agardhii*–specific *mcy*E gene copy number and the total concentration of MCs was evaluated using the Pearson correlation coefficient. Statistical analysis was performed using Statistica 7.0.

## 3. Results

### 3.1. Environmental Variables in the Studied Lakes

The studied lakes were assigned to the eutrophic water body type with high total phosphorus, nitrogen and chl-*a* concentrations and low water transparency in summer (Table 2). The surface water temperature during the study period was similar in both lakes (from 16.8 to 17.6 °C), with an average of 21.0–21.5 °C in summer. Secchi depth was up to 2.4 times higher in Lake Širvys compared to Lake Jieznas. However, average total nutrient and chl-*a* concentrations were up to 1.8 times higher in Lake Jieznas compared to Lake Širvys.

### 3.2. Seasonal Variation of the Cyanobacteria Community in Mono- and Polidominant Temperate Lakes

**Figure 1:**
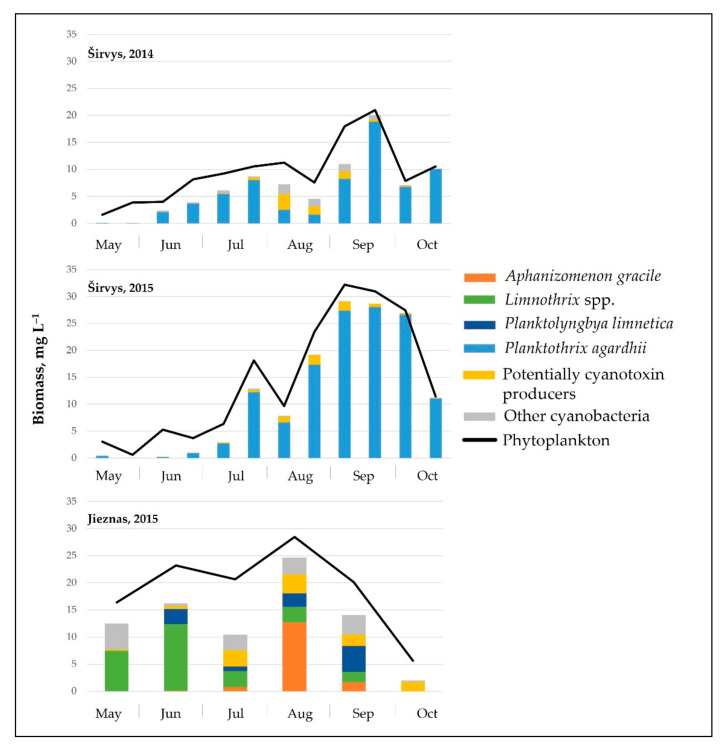
Seasonal variation in the biomass of potentially toxin-producing cyanobacteria species in the studied lakes.

The studied lakes differed significantly in the structure of the dominant cyanobacteria species. In the monodominant Lake Širvys, the potentially hepatotoxic *P*. *agardhii* was the single dominant species in the phytoplankton during the two years studied, accounting for up to 28.1 mg L^−1^ biomass, with the highest values recorded in autumn (up to 97% of the total biomass of cyanobacteria) (Figure 1). The polidominant Lake Jieznas was dominated by the potentially neurotoxins producing *Aphanizomenon gracile* in August (up to 12.7 mg L^−1^, 45% of the total cyanobacterial biomass). *Limnothrix planctonica* and *L*. *redekei* prevailed in May–June (up to 12.3 mg L^−1^) and *Planktolyngbya limnetica* in September (up to 4.8 mg L^−1^). The dominant species in one lake were also present in another studied lake but accounted for only a small portion of the biomass. Furthermore, 14 others potential cyanotoxin producers, mainly species from *Microcystis*, *Dolichospermum*, *Aphanizomenon*, *Raphidiopsis, Woronichinia* genera, were detected with negligible biomass (Figure 1).

### 3.3. Diversity and Amount of Cyanometabolites in Studied Lakes

The same groups of cyanometabolites were found in the studied mono- and polidominant lakes: MCs, STX, ATX-a and other NRPs; however, they varied in diversity and quantities.

#### Seasonal Variation of Cyanometabolites in Lakes

**Figure 2:**
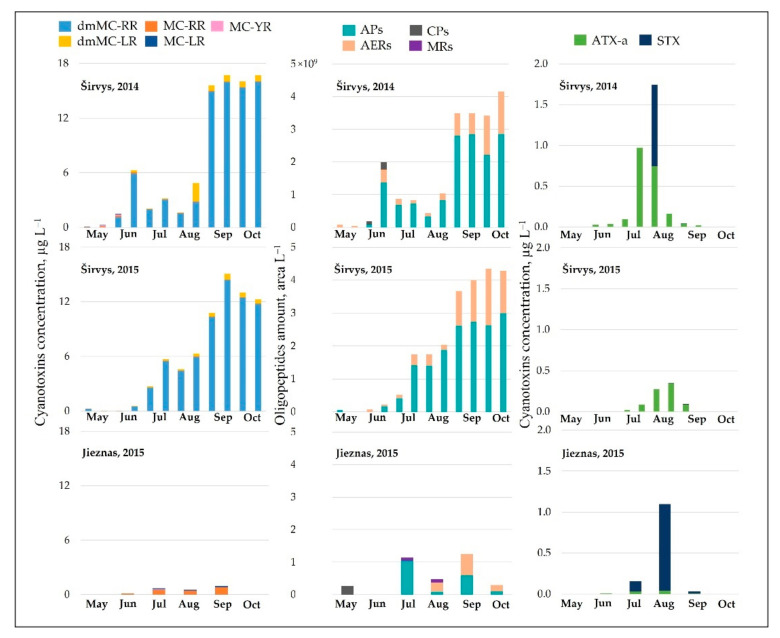
The variation of intracellular cyanometabolites in the field samples of Širvys (monodominant) and Jieznas (polidominant) lakes.

MCs and other NRPs. In the monodominant Lake Širvys, MCs were detected throughout the study period from May to October, with the highest concentrations occurring in autumn (10.76–16.74 µg L^−1^) (Figure 2). In polidominant Lake Jieznas, MCs values were rather low and were detected in June–September (0.15–0.96 µg L^−1^). Three microcystin variants MC-LR, MC-YR, MC-RR were common and present in both lakes. MC-RR dominated in the polidominant lake comprising 77.7 ± 6.3%, followed by MC-LR with 19.0 ± 2.3% of the total concentration of MC in June–September. Meanwhile, dmMC-RR and dmMC-LR were detected only in the monodominant lake, where dmMC-RR was the dominant variant contributing an average of 87.2 ± 21.6% and frequently exceeding 95%, followed by dmMC-LR with 6.4 ± 8.6% of the total MCs concentration in the summer–autumn months.

NRPs concentration was 3.1 times higher in the monodominant Lake Širvys than in the polidominant Lake Jieznas during June–October. The peak concentrations (3.42–4.35 × 10^9^ area L^−1^) were detected in the monodominant lake in autumn. In the polidominant lake, the two peaks (1.13 × 10^9^ and 1.25 × 10^9^ area L^−1^) were found in July and September, respectively (Figure 2). In the monodominant lake, the dynamics of MCs and NRPs coincided, and the concentration increased from spring to autumn, with the highest values in September and October and stable structure of the detected cyanometabolites groups. The dominant group of oligopeptides was APs followed by AERs and comprised 76.6% and 30.3% of the total amount of NRPs, respectively. In contrast, the dynamics and structural trends of MCs and other NRPs differed in the polidominant lake. In May, only CPs were present. APs dominated in July and AERs in August, and accounted for 91.1% and 58.7%, respectively. In autumn months (September–October), the group of AERs dominated with 52.4% and 66.8%, respectively, the other part consisted of APs.

**Figure 3:**
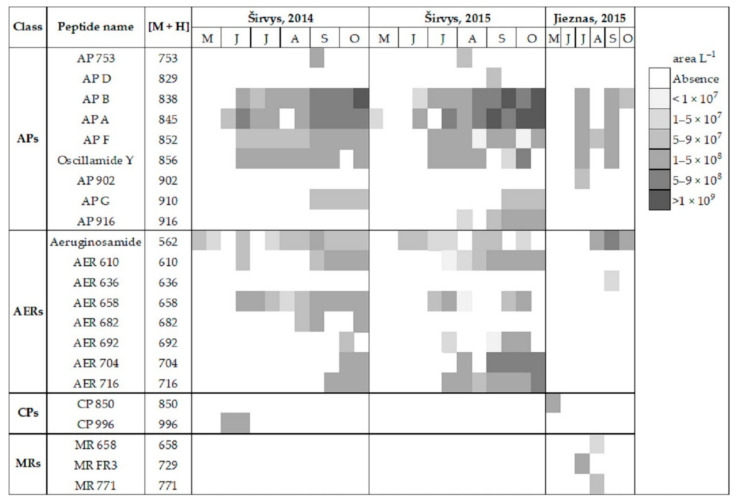
Profile of non-ribosomal peptides (NRPs) in the water samples of Širvys (monodominant) and Jieznas (polidominant) lakes.

In the studied lakes, these common APs (A, B, F and Oscillamide Y) and AERs (aeruginosamide) were found (Figure 3). In the monodominant Lake Širvys, the greatest diversity of APs and AERs groups was detected from June to October. The most frequent oligopeptides in the monodominant lake were APs (A, B, F, Oscyllamide Y), AERs (aeruginosamide, AER 610, 658) occurred from June to October, and the highest concentrations of AP A and AP B (maximum 1.10 × 10^9^ and 1.32 × 10^9^ area L^−1^, respectively) were found from September to October. However, the diversity of NRPs in the polidominant Lake Jieznas was much lower. The most diverse profile of the APs group was found in July and September and consisted almost entirely of common oligopeptides. In both lakes, specific oligopeptides were mostly found only once or for a short period of time, except for AP G, AER 610, AER 658 and AER 716 in the monodominant lake (Figure 3).

Neurotoxins. Among this group, STX dominated in the polidominant Lake Jieznas, while ATX-a predominated in the monodominant Lake Širvys. Anatoxin-a was detected in both lakes from June to September. The average ATX-a concentration was 9.7 times higher in the monodominant lake than in the polidominant lake during the summer–autumn. The maximum values were detected in both lakes in July–August, reaching 0.97 µg L^−1^ and 0.04 µg L^−1^, respectively (Figure 2). The average STX concentration was 4.9 times higher in the polidominant lake than in the monodominant lake summer–autumn months. In one case, a maximum concentration of 1.00 µg L^−1^ was found in the monodominant lake (August 2014), while the other values were negligible. In contrast, STX was detected in the polidominant lake from July to September with a similar peak concentration of 1.06 µg L^−1^ (Figure 2). Cylindrospermopsin was not detected in environmental samples or isolates.

### 3.4. Cyanometabolites in Isolates of Cyanobacteria

A total of 248 isolates isolated from two studied lakes and belonging to 15 cyanobacteria species were analysed for the presence of cyanotoxins and other NRPs (Tables S1–S3).

Producers in Chroococcales and Oscillatoriales. All five *Microcystis viridis* isolates produced the highest amount of MCs on average of 0.93 ± 0.36 µg mg^−1^ MCs in freeze-dried biomass, followed by one of the six *M*. *aeruginosa* isolates (0.41 µg mg^−1^ freeze-dried biomass) and negligible concentrations in two of three isolates of *M*. *flos*-*aquae* (Table S1). Five *M*. *wessenbergii* isolates tested did not produce MCs. *M*. *flos*-*aquae* was distinguished by producing only MC-RR. Isolates of both *M*. *aeruginosa* and *M*. *viridis* species were characterized by dominating MC-LR with an average of 55.6% and 56.9% and prevailed by MC-YR (26.6% and 23.6%), also followed by MC-RR (14.5%, 9.3%) and dmMC-LR (3.4% and 10.2%), respectively. The great variety of MCs was detected in *M*. *aeruginosa* and *M*. *viridis*, while the lowest was in *M*. *flos*-*aquae*. The average MCs concentration of 0.99 ± 0.57 µg mg^−1^ freeze-dried biomass in seven of eight *Planktothrix agardhii* isolates coincided with total MCs amount found in *Microcystis viridis* isolates. The profile of MCs in *P*. *agardhii* isolates was characterized by the dominant dmMC-RR variant, which accounted for 60.3% on average (up to 93.5%), followed by dmMC-LR (23.0%), MC-RR (14.4%) and MC-YR (2.3%) (Table S2).

The profile of other NRPs was more diverse among *Microcystis* spp. than in the *P*. *agardhii* isolates. *M*. *aeruginosa* and *M*. *viridis* mainly synthesized CPs (average 72.2% and 75.0%, respectively), followed by APs (17.8%) and AERs (25.0%), respectively (Table S1). However, in *M*. *flos*-*aquae* isolates, AERs dominated at 66.7%, while APs (20.3%) prevailed. NRPs were detected in all six *P*. *agardhii* isolates tested (Table S2). In contrast to *Microcystis* spp., on average APs dominated and accounted for 70.6% (three isolates produced only APs), AERs prevailed at 17.3%, followed by CPs with 12.1% in *P*. *agardhii* isolates. At the same time *M*. *aeruginosa* (1 isolate), *M*. *flos*-*aquae* (1 isolate) and *P*. *agardhii* (4 isolates) synthesized APs oligopeptides commonly detected in environmental samples, such as A, B, F, Oscillamide Y. In addition, aeruginosamide was detected in one *M*. *flos*-*aquae* isolate. In *P*. *agardhii* isolates, the group of APs was detected in all isolates and showed the highest internal diversity of APs and AERs. Although the profiles of NRPs varied among *P*. *agardhii* isolates, oligopeptides that could be synthesized by several isolates were frequently detected. *Limnothrix planctonica* and *Planktolyngbya limnetica* did not synthesize MCs and other NRPs.

Producer in Nostocales. In total, nineteen isolates of *Aphanizomenon gracile* (10 isolates), *Cuspidothrix issatschenkoi* (5 isolates), *Anabaenopsis* cf. *elenkinii*, (2 isolates) and *Dolichospermum crassum* (2 isolates) were screened for NRPs. However, only three isolates of *A*. *gracile* were able to synthesize oligopeptides (Table S3). On average, APs dominated and accounted for 86.8% of the total NRPs, which was also characterized by the greatest variety in the isolates, followed by MRs with 8.3% and CPs with 4.9%. The isolates produced common oligopeptides such as AP A (2 isolates), AP B (3 isolates), AP F (3 isolates), which were identified in *M*. *aeruginosa*, *M*. *flos*-*aquae* and *P*. *agardhii*. These isolates were also characterized by specific oligopeptides. Single oligopeptides belonging to the group CPs and MRs were present, but the group of AERs was not detected. Cyanotoxins and/or other NRPs were not found in the isolates of *Anabaenopsis* cf. *elenkinii*, *Cuspidothrix issatschenkoi*, *Dolichospermum crassum*, *D*. *lemmermannii*, *Raphidiopsis mediterranea*, *Sphaerospermopsis aphanizomenoides*.

### 3.5. Planktothrix agardhii mcyE Gene in Isolates and Environmental Samples from the Monodominant Lake

**Figure 4:**
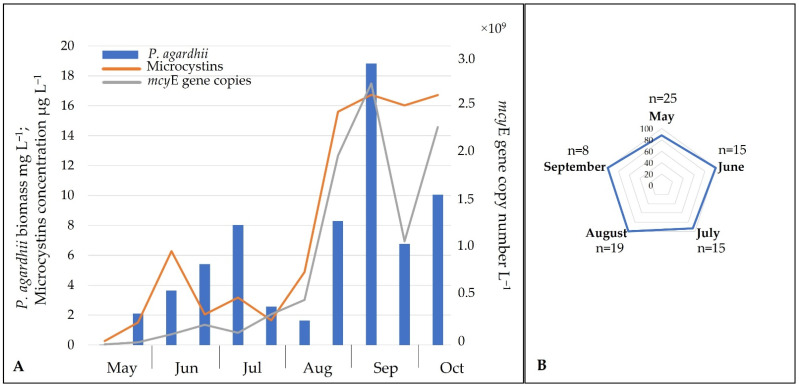
(**A**)—Dynamics of *Planktothrix agardhii* (expressed by biomass), concentration of microcystins and *mcy*E gene copy number in the environmental samples of Lake Širvys (monodominant) in 2014. (**B**)—Relative number (%) of toxigenic isolates of *P*. *agardhii* (n—number of isolates tested).

The qPCR analysis showed that the highest copy number 26 × 10^9^ L^−1^ of the *mcy*E gene specific for *Planktothrix* was detected in the field samples in September and coincided with the highest biomass of *P*. *agardhii* and MCs concentration (Figure 4A). A strong correlation was observed between *P*. *agardhii*-specific *mcy*E gene copy number and total MCs concentration in field samples (r = 0.85, *p* < 0.05). Additionally, *P*. *agardhii* isolates analysis using conventional PCR revealed that 95.1% of the 82 isolates examined contained *mcy*E synthetase gene (Table S2). The percentage of toxic isolates increased slightly toward the summer–autumn months (Figure 4B).

## 4. Discussion

### 4.1. Structure and Biomass of Cyanobacteria in Mono- and Polidominant Shallow Eutrophic Lakes

Cyanobacteria dominated over other algal groups in the phytoplankton of the studied lakes. According to WHO [40] guidelines for monitoring and management of cyanobacteria in recreational waters, cyanobacteria biomass in the monodominant Lake Širvys reached alert level 1 (4–8 mg L^−1^) since July, while in the polidominant Lake Jieznas it exceeded alert level 1 already in May. The maximum total biomass of cyanobacteria in the studied lakes was similar, but the species composition and dynamics of cyanobacteria varied between the two lakes (Figure 1). The predominant filamentous cyanobacteria species from the genera *Aphanizomenon*, *Planktothrix*, *Limnothrix* and *Planktolyngbya* are characteristic to nutrient–rich lakes [41] and were dominant in the studied lakes.

In the monodominant lake, *Planktothrix agardhii* outcompeted other harmful cyanobacteria throughout the vegetation season. This species accounted for a significant amount of the biomass in summer and autumn (up to 17.4 mg L^−1^ and 28.1 mg L^−1^, respectively) and accounted for 93.3% of the total cyanobacteria biomass (Figure 1). In European eutrophic freshwaters, *P*. *agardhii* formed the highest biomass with up to 54–70 mg L^−1^ (45–100% of total phytoplankton biomass [5,42]), and even 600 mg L^−1^ in autumn [43]. This species is characterized by recurrent dominance in shallow eutrophic and hypertrophic lakes [44,45].

Cyanobacteria assemblage in the polidominant lake was characterized by the dynamics of a few predominant species that prevailed over different period (Figure 1). The peak cyanobacterial biomass was observed in August when *A*. *gracile* was the dominant species (Figure 1). *Limnothrix* spp. dominated in May–June and prevailed in July–August, while *Planktolyngbya limnetica* prevailed in August and dominated in September. According to Komárek [29], *A*. *gracile* is a frequent species, but rarely forms blooms in temperate regions of the Northern Hemisphere. In European lakes, *A*. *gracile* was the dominant species and formed a similar biomass of up to 15 mg L^−1^ [12,46]. The highest values were up to 33 mg L^−1^ (max 80% of total phytoplankton biomass; [47]). A similar prevailing species composition (*Aphanizomenon gracile*, *Planktolyngbya limnetica*, *Limnothrix redekei*) was also characteristic to the shallow eutrophic lake in Poland [48]. The dominant *A*. *gracile* may also coexist with the species *P*. *agardhii* and *Microcystis aeruginosa* in various complexes [49].

### 4.2. Cyanometabolite Producers

Toxin production varies spatially and temporally during bloom [50], depending on the ability of bloom-forming cyanobacteria to produce cyanometabolites. Therefore, it is critical to identify toxin-producing cyanobacterial species to predict cyanotoxin diversity and concentration in the water body. Fifteen potentially toxic species and a total of 248 isolates isolated from the studied lakes were analysed for the presence of cyanometabolites to explain their seasonal variation in environmental samples.

Microcystins accounted for a significant portion of the cyanotoxins identified in the monodominant Lake Širvys. Analysis of *Planktothrix agardhii* isolates and environmental samples revealed that this dominant species was solely responsible for the production of MCs and the other NRPs. 95.1% of the over than eighty *P*. *agardhii* isolates possessed a gene responsible for the production of MCs. The seasonal dynamics of *P*. *agardhii mcy*E synthetase gene copy number in the environmental samples also corresponded to the biomass of the species and MCs concentration (Figure 4A). A high positive correlation between *P*. *agardhii* biomass and total MCs concentration in other European lakes was observed [5,12,51]. This is also consistent with the data of Kurmayer et al. [52] and Yéprémian et al. [43], where 88% and 52% of toxic *P*. *agardhii* isolates were detected, respectively. Briand et al. [10] also confirmed a significant correlation between *P*. *agardhii mcy*A copy number and MC values. The proportion of prevailing dmMC-RR (average 87.2%) and dmMC-LR (average 6.4%) in the studied monodominant lake was similar to the proportion of MCs variants detected in *P*. *agardhii* isolates from the same water body (60% of dmMC-RR and 23% of dmMC-LR). Consistent with the results of Schwarzenberger et al. [53], *P*. *agardhii* isolates contained higher dmMC-RR concentrations (up to 40-fold) compared to dmMC-LR. *P*. *agardhii* is a well-documented producer of various cyanometabolites (MC-RR, MC-YR, dmMC-RR, dmMC-LR, MC-LR, Asp3 MC-LR, APs B and F) [53,54,55].

Janssen [22] highlighted that MCs typically occur together with other bioactive NRPs and never alone. In the current study, the production of MCs was associated with a higher relative amount of NRPs. The relative proportion of other NRPs was similar in environmental samples and *P*. *agardhii* isolates; for example, APs comprised 70.6% in samples and 76.7% in isolates, whereas AERs consisted of 17.3% and 30.3%, respectively. Anabaenopeptins are frequently detected in lakes dominated by *P*. *agardhii* (e.g., Grabowska et al. [5]). Schwarzenberger et al. [53] also found that *P*. *agardhii* produced other NRPs in addition to MCs, with predominant AERs (70%), APs (up to 21.6%) and planktocyclins (50.5%) varying among isolates. In general, our analysis confirmed the high potential of *P*. *agardhii* to be toxic and to synthesize a wide range of MCs variants and other oligopeptides. Compared to *Planktothrix*, *Microcystis* species generally found to have a greater variety of NRPs [56,57].

The total average concentration of cyanotoxins was 9.5 times higher in the monodominant lake than in the polidominant lake (Table 3). Moreover, MCs prevailed over neurotoxins in the monodominant lakes, whereas MCs and neurotoxins formed similar concentrations in the polidominant lake. NRPs were detected only in *Aphanizomenon gracile* from the three dominant species in the lake; the co-dominant *Planktolyngbya limnetica* and *Limnothrix* spp. were not producers of cyanometabolites. The peak biomass of *A*. *gracile* coincided with the highest STX concentration in the environmental samples, suggesting that this species is a possible toxin producer (Figure 2 and Figure 3). According to Karosienė et al. [32], the species was confirmed as a producer of STX in the studied lakes. Only 11 of 63 *A*. *gracile* isolates tested (17.5%) were able to produce STX with concentrations ranging from 0.54 × 10^−4^ to 4.67 × 10^−4^ µg mg^−1^ freeze-dried biomass [32]. The current study revealed evident differences in the ability to synthesize cyanotoxins between species. Hepatotoxin producer *P*. *agardhii* had a higher ability to produce MCs (average concentration 0.99 µg mg L^−1^ freeze-dried biomass) and included a relatively higher number of toxic isolates (95.1%) compared to the saxitoxin producer *A*. *gracile* [32]. Similarly, Casero et al. [58] also detected a low ability and capacity of *A. gracile* to synthesize STX (11.1% of STX synthesizing isolates at a concentration of 0.17–0.42 µg equivalent STX mg^−1^ DW).

*Microcystis* spp. isolates showed great potential to produce MCs and contributed to toxic blooms in the studied polidominant lake. Especially MC-RR and MC-LR were found in *M*. *viridis* (14.5% and 55.6%, respectively), *M*. *flos*-*aquae* (9.3% and 56.9%, respectively) and 100% of MC-RR was determined in *M*. *aeruginosa* isolates. In contrast to our results, Via-Ordorika et al. [59] found a low percentage (17%) of *M*. *viridis* colonies containing *mcy* gene and MCs, whereas it was moderate for *M*. *flos*-*aquae* (50%) and high for *M*. *aeruginosa* (72%). *Microcystis* spp. are associated with the production of more than one variant of MCs, e.g., MC-LR, MC-RR, MC-YR, MC-D-Asp3-LR, MC-RR [60,61]. *Planktothrix agardhii* and *Microcystis* are the best–known genera for their ability to produce MCs [62] with similar maximum concentrations determined as high as 4–4.5 µg mg DW^−1^ [63,64]. Compared to the *Planktothrix*–dominated lake, the amount of NRPs in the polidominant lake was 2.8 times lower and their profile varied significantly throughout the season, possibly reflecting changes in the cyanobacteria community. The study showed that of all the dominants found in the polidominant lake, only non–STX–producing isolates of *A*. *gracile* were capable of synthesizing NRPs. APs was dominant oligopeptide group, followed by MRs and CPs.

## 5. Conclusions

The study revealed differences in the composition of the predominant cyanobacteria species, but the total biomass of potential toxin producers was similar in the studied lakes. *Planktothrix agardhii* displaced other harmful cyanobacteria and was the only dominant species in Lake Širvys. In contrast, the potential cyanotoxins producers *Aphanizomenon gracile*, *Limnothrix* spp. and *Planktolyngbya limnetica* co-dominated in Lake Jieznas at different period. Cyanometabolites of the same groups (MCs, other NRPs, ATX-a and STX) were found in studied freshwaters; however, total concentration of cyanotoxins was up to 9.5 times higher and the amount of NRPs was up to 2.8 times higher in the monodominant lake. The biomass of *Planktothrix agardhii* was up to 28.1 mg L^−1^, the species had over 95% of the toxic individuals in the population and was basically responsible for the synthesis of the predominant cyanometabolites (dmMC-RR, dmMC-LR, APs, and AERs), indicating an increased risk for recreational activities in the monodominant lake. Only *Aphanizomenon gracile* was able to synthesise cyanometabolites in the polidominant lake, while no toxic compounds were detected in the co-dominant species *Limnothrix planctonica* and *P*. *limnetica*. The non-dominant species *Microcystis aeruginosa*, *M*. *flos*-*aquae*, and *M*. *viridis* contributed to the total amount of cyanometabolites, but in general the concentration of cyanotoxins did not pose a risk to bathers in the polidominant lake. The greater diversity of cyanometabolites detected in the cyanobacteria isolates than in the environmental samples suggests a possible variation in the cyanometabolite profile.

## Figures and Tables

**Table 1:** Morphometric characteristics of the studied lakes and land use of their catchment area.

Morphometric Data	Širvys	Jieznas
Coordinates	54°59′16.27″, 25°12′54.13″	54°35′33.67″, 24°10′48.95″
Altitude (m)	125.8	95.8
Max depth (m)	4.5	4.4
Mean depth (m)	1.4	2.8
Catchment area consisted of (%) *:		
Natural biotopes	47.4	4.0
Agriculture	48.3	83.6
Villages	4.3	12.4

*—The data was provided from Balevičius et al. [24].

**Table 2:** Physicochemical, biological variables and trophic status of the lakes Širvys and Jieznas during the study period. The data are presented as average ± SD.

Variable	Širvys		Jieznas 2015
	2014	2015	
Water temperature, °C	17.6 ± 5.5	16.8 ± 5.2	16.9 ± 5.3
Secchi depth, m	1.15 ± 0.60	1.30 ± 0.70	0.55 ± 0.10
pH	8.3 ± 0.3	8.3 ± 0.3	8.3 ± 0.2
Conductivity, µS cm^−1^	444.1 ± 7.2	445.4 ± 8.5	441.7 ± 10.6
Dissolved oxygen, mg L^−1^	10.4 ± 2.0	10.5 ± 2.8	10.9 ± 1.5
TP, mg P L^−1^	0.034 ± 0.012	0.035 ± 0.017	0.059 ± 0.026
TN, mg N L^−1^	1.25 ± 0.2	1.23 ± 0.3	1.84 ± 0.3
Chlorophyll-*a*, µg L^−1^	35.8 ± 17.0	34.5 ± 15.8	61.4 ± 11.2
Trophic status *	eutrophic	eutrophic	eutrophic

*—trophic state was assessed according to the general trophic classification of Wetzel [38] (modified from Vollenweider [39].

**Table 3:** Summarized data on cyanobacteria, cyanotoxins and other NRPs of mono- and polidominant lakes.

Variables	Monodominant Lake Širvys	Polidominant Lake Jieznas
Number of dominants	1	3
Number of species potential cyanotoxins producers	14	14
Average (±SD) biomass of potential cyanotoxins producers, mg L^−1^	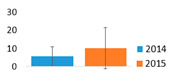	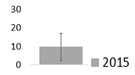
Average (±SD) total concentration of cyanotoxins, µg L^−1^	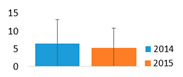	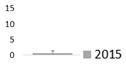
Average concentration of MCs, ATX, STX, µg L^−1^	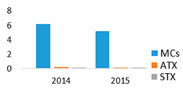	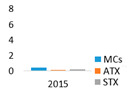
Average NRPs amount, area L^−1^	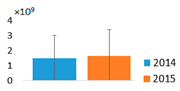	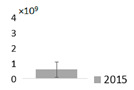
Isoforms of MCs detected in the field samples	dmMC-RR, MC-RR, MC-YR, dmMC-LR, MC-LR	MC-RR, MC-YR, MC-LR
NRPs groups detected in field samples, area L^−1^	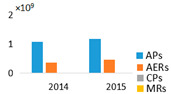	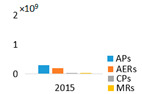
Common NRPs	APs (A, B, F, Oscillamide Y), AERs (aeruginosamide)	
Specific NRPs	APs (753, D, G, 916)	APs (902), AERs (636)
	AERs (658, 682, 692, 704, 716), CPs (996)	CPs (850), MRs (658, FR3, 771)

## Data Availability

Not applicable.

## Supplementary Materials

The following supporting information can be downloaded at: https://www.mdpi.com/article/10.3390/ijerph192215341/s1, Table S1. Cyanotoxins and other non-ribosomal peptides (NRPs) profile in Chroococcales cyanobacteria isolates. Table S2. Cyanotoxins and other non-ribosomal peptides (NRPs) profile in Oscillatoriales cyanobacteria isolates. Table S3. Cyanotoxins and other non-ribosomal peptides (NRPs) profile in Nostocales cyanobacteria isolates.
