# Supplementary material for: Cyanobacteria and Their Metabolites in Mono- and Polidominant Shallow Eutrophic Temperate Lakes
Source: Int J Environ Res Public Health. 2022 Nov 20;19(22):15341. doi: 10.3390/ijerph192215341 (PMC9690872; doi:10.3390/ijerph192215341)
Supplement: Supplementary file 1 [file ijerph-19-15341-s001.zip › ijerph-1977624-supplementary.pdf]

Table S1. Cyanotoxins and other non-ribosomal peptides (NRPs) profile in Chroococcales cyanobacteria isolates.

Concentration	Class	Cyanometabolite	[M + H]	*Microcystis aeruginosa*						*Microcystis flos-aquae*			*Microcystis viridis*					*Microcystis wessenbergii*	
				NRC_JIE/B6-07	NRC_JIE/B11-05	NRC_JIE/C6-07	NRC_JIE/D7-07	NRC_JIE/F9-06	NRC_SIR/D7-09	NRC_JIE/E11-05	NRC_SIR/D11-09	NRC_SIR/E10-09	NRC_JIE/C4-08	NRC_JIE/D8-06	NRC_JIE/F10-05	NRC_JIE/G4-08	NRC_JIE/G10-05	5 isolates JIE	
Cyanotoxin, µg mg^{-1} freeze-dried biomass	MCs	Asp3 MC-LR																	
		Asp3 MC-RR																	IDA*
		dmMC-LR							0.014										
		dmMC-RR											0.063	0.088	0.092	0.121	0.066		
		MC-LR							0.230				0.766	0.617	0.427	0.497	0.311		
		MC-RR							0.060		6.7×10^{-5}	0.002	0.086	0.106	0.006	0.129	0.084		
		MC-YR							0.110				0.599	0.146	0.176	0.165	0.116		
		MC-WR																	
	ATX-a																		
	STX																		
Peptides area g^{-1} freeze-dried weight	APs	Oscillamyde Y	589																
		AP 767	767																
		AP B	838																Absence
		AP A	845																$< 1 \times 10^7$
		AP F	852																$1–9 \times 10^7$
	AERs	Aeruginosamide	562																$1–9 \times 10^8$
		AER 576	576																$1–9 \times 10^9$
		AER 582	582																$1–9 \times 10^{10}$
		AER 610	610																$>1 \times 10^{11}$
		AER 622	622																
		AER 636	636																
		AER 649	649																
		AER 748	748																
		AER 763	763																
	CPs	CP 936	936																
		CP 944	944																
		CP 958	958																
		CP 975	975																
		CP 982	982																
		CP 993	993																
		CP 995	995																
		CP 997	997																
		CP 1011	1011																
		CP 1014	1014																
		CP 1022	1022																
		CP 1027	1027																
		CP 1062	1062																
	MRs	MR FR6	741																
		MR 785	785																
		MR 799	799																

*IDA – information-dependent acquisition method (qualitative data); JIE – isolates from Lake Jieznas; SIR – isolates from Lake Širvys.

Table S2. Cyanotoxins and other non-ribosomal peptides (NRPs) profile in Oscillatoriales cyanobacteria isolates.

Concentration	Class	Cyanometabolite	[M + H]	*Limnothrix planctonica*	*Planktolyngbya limnetica*		*Planktothrix agardhii*									
				7 isolates JIE	3 isolates JIE	6 isolates SIR	NRC_JIE/C9-07	NRC_JIE/E9-07	NRC_SIR/C11-09	NRC_SIR/C12-09	NRC_SIR/E8-07	NRC_SIR/F5-07	NRC_SIR/G5-05	NRC_SIR/G6-05	72 isolates SIR	4 isolates SIR
Cyanotoxin, μg mg^{-1} freeze-dried biomass	MCs	Asp3 MC-LR													+	-
		Asp3 MC-RR														
		dmMC-LR							0.146	0.396	0.169	0.193	0.071	0.210		
		dmMC-RR							0.892		1.013	1.105	1.357	1.154		
		MC-LR														
		MC-RR					7.4×10^{-5}				0.002	0.001	0.002	0.001		
		MC-YR									0.032	0.083	0.022	0.084		
		MC-WR														
	ATX-a														n.a.	n.a.
	STX														n.a.	n.a.
Peptides area g^{-1} freeze-dried weight	APs	AP 835	835				n.a.	n.a.							n.a.	n.a.
		AP B	838				n.a.	n.a.							n.a.	n.a.
		AP A	845				n.a.	n.a.							n.a.	n.a.
		AP F	852				n.a.	n.a.							n.a.	n.a.
		Oscillamide Y	859				n.a.	n.a.							n.a.	n.a.
		AP 944	944				n.a.	n.a.							n.a.	n.a.
	AERs	AER 610	610				n.a.	n.a.							n.a.	n.a.
		AER 705	705				n.a.	n.a.							n.a.	n.a.
		AER 718	718				n.a.	n.a.							n.a.	n.a.
		AER 738	738				n.a.	n.a.							n.a.	n.a.
	CPs	CP 964	964				n.a.	n.a.							n.a.	n.a.
		CP 982	982				n.a.	n.a.							n.a.	n.a.
		CP 996	996				n.a.	n.a.							n.a.	n.a.
	MRs						n.a.	n.a.							n.a.	n.a.

Legend:
- IDA*
- Absence
- < 1 × 10^{7}
- 1–9 × 10^{7}
- 1–9 × 10^{8}
- 1–9 × 10^{9}
- 1–9 × 10^{10}
- >1 × 10^{11}

*IDA – information-dependent acquisition method (qualitative data); JIE – isolates from Lake Jieznas; SIR – isolates from Lake Širvys.

Table S3. Cyanotoxins and other non-ribosomal peptides (NRPs) profile in Nostocales cyanobacteria isolates.

Concentration	Class	Cyanometabolite	[M + H]	*Anabaenopsis* cf. *elenkinii*	*Aphanizomenon gracile*						*Aphanizomenon flos-aquae*	*Cuspidothrix issatschenkoi*	*Dolichospermum crassum*		*Dolichospermum lemmermannii*	*Raphidiopsis mediterranea*	*Sphaerospermopsis aphanizomenoides*
				2 isolates JIE	29 isolates JIE	NRC_SIR/D5-09	NRC_SIR/E9-08	NRC_SIR/G5-09	7 isolates SIR	27 isolates SIR	3 isolates SIR	5 isolates JIE	39 isolates SIR	2 isolates SIR	NRC_SIR/C5-05	2 isolates SIR	12 isolates JIE
Toxin, μg mg^{-1} freeze-dried biomass	MCs												n.a.		n.a.		
	ATX-a																
Peptides area g^{-1} freeze-dried weight	APs	AP 810	810		n.a.			1–9 × 10^{9}		n.a.	n.a.		n.a.		n.a.	n.a.	n.a.
		AP 815	815		n.a.			1–9 × 10^{10}		n.a.	n.a.		n.a.		n.a.	n.a.	n.a.
		AP 824	824		n.a.	1–9 × 10^{9}				n.a.	n.a.		n.a.		n.a.	n.a.	n.a.
		AP D	829		n.a.	1–9 × 10^{10}		1–9 × 10^{10}		n.a.	n.a.		n.a.		n.a.	n.a.	n.a.
		AP B	838		n.a.	1–9 × 10^{10}	1–9 × 10^{10}	1–9 × 10^{10}		n.a.	n.a.		n.a.		n.a.	n.a.	n.a.
		AP A	845		n.a.		1–9 × 10^{10}	1–9 × 10^{9}		n.a.	n.a.		n.a.		n.a.	n.a.	n.a.
		AP F	852		n.a.	1–9 × 10^{10}	1–9 × 10^{9}	1–9 × 10^{10}		n.a.	n.a.		n.a.		n.a.	n.a.	n.a.
		Oscillamide Y	859		n.a.					n.a.	n.a.		n.a.		n.a.	n.a.	n.a.
		AP 870	870		n.a.	1–9 × 10^{9}				n.a.	n.a.		n.a.		n.a.	n.a.	n.a.
		AP 999	999		n.a.		1–9 × 10^{10}			n.a.	n.a.		n.a.		n.a.	n.a.	n.a.
	AERs				n.a.					n.a.	n.a.		n.a.		n.a.	n.a.	n.a.
	CPs	CP 843	843		n.a.			1–9 × 10^{10}		n.a.	n.a.		n.a.		n.a.	n.a.	n.a.
		CP 988	988		n.a.		1–9 × 10^{10}			n.a.	n.a.		n.a.		n.a.	n.a.	n.a.
	MRs	MR 828	828		n.a.		1–9 × 10^{10}			n.a.	n.a.		n.a.		n.a.	n.a.	n.a.

Legend
Absence
< 1 × 10^{7}
1–9 × 10^{7}
1–9 × 10^{8}
1–9 × 10^{9}
1–9 × 10^{10}
>1 × 10^{11}

JIE – isolates from Lake Jieznas; SIR – isolates from Lake Širvys.
